# The Vast Potential of ChatGPT in Pediatric Surgery

**DOI:** 10.2196/66453

**Published:** 2024-11-18

**Authors:** Ran Tang, Shi-qin Qi

**Affiliations:** 1 Department of Pediatric Surgery Anhui Provincal Children's Hospital Hefei China

**Keywords:** ChatGPT, pediatric, surgery, artificial intelligence, AI, diagnosis, surgeon

We came across the publication “The Diagnostic Ability of GPT-3.5 and GPT-4.0 in Surgery: Comparative Analysis” [1] in the *Journal of Medical Internet Research* by Liu and colleagues. As highlighted in this article, the diagnostic prowess of artificial intelligence (AI) systems like GPT-4 continues to advance rapidly, showcasing remarkable improvements over earlier models [2]. These findings emphasize the potential of AI in diagnosing colorectal cancer and provide insights into its possible applications in pediatric surgery.

Pediatric surgery, distinct from adult surgery, presents unique challenges: children’s developmental and anatomical differences, a limited disease spectrum, and the delicate balance between surgical trauma and recovery. Moreover, communication barriers with young patients often place a greater burden on clinicians for accurate diagnosis and effective care planning. In areas like Anhui Province, China, which is home to over 60 million people but served by only one pediatric center, nonspecialist doctors must often perform surgeries, heightening the risk of complications.

The application of AI in pediatric surgery has begun to bridge these gaps. GPT-4’s capabilities in multimodal data integration, such as processing medical records, images, and lab results, offer substantial potential in preoperative planning, providing tailored insights that pediatric surgeons can use to navigate complex cases. Furthermore, GPT-4’s utility in enhancing surgical education is noteworthy. Through integration with 3D models and its conversational abilities, AI can offer virtual simulations and detailed anatomical explanations, greatly benefiting students and junior doctors who need hands-on experience in congenital anomalies and tumor resections.

Postoperative care is another critical area where AI can provide substantial support. As children are less able to articulate their needs, GPT-4’s real-time data processing and monitoring abilities can aid in identifying early signs of complications and offer personalized recovery plans. In resource-limited regions, AI can facilitate remote collaboration, linking local hospitals with specialists in pediatric centers, ultimately improving care outcomes.

Liu et al [1] emphasized the potential of AI in refining diagnostic tools. However, it is important to note that AI lacks the clinical intuition required in many pediatric surgery cases. While AI systems like GPT-4 provide valuable assistance, they cannot replace the clinical judgment of experienced pediatric surgeons, especially when dealing with complex or nonstandard scenarios.

In conclusion, GPT-4 offers a promising future for pediatric surgery, from improving diagnostic accuracy and surgical education to enhancing postoperative care. However, its limitations in clinical decision-making highlight the need for careful integration of AI technologies into practice, with clear guidelines to ensure ethical and effective use. With proper regulation, GPT-4 can become an indispensable tool in pediatric surgery, particularly in regions with limited access to specialized care.

## Abbreviations

AI: artificial intelligence
